# C7ORF41 Regulates Inflammation by Inhibiting NF-*κ*B Signaling Pathway

**DOI:** 10.1155/2021/7413605

**Published:** 2021-01-06

**Authors:** Jinping Zhou, Quan Zhou, Tan Zhang, Jingyi Fan

**Affiliations:** ^1^Department of Pediatrics, Zhongnan Hospital of Wuhan University, Donghu Road 169, Wuhan, HuBei 430072, China; ^2^College of Life Sciences, Wuhan University, Wuhan 430072, China

## Abstract

Inflammation is an important biological process for eliciting immune responses against physiological and pathological stimuli. Inflammation must be efficiently regulated to ensure homeostasis in the body. Nuclear factor-kappa B (NF-*κ*B) signaling is crucial for inflammatory and immune responses. Aberrant activation of NF-*κ*B signaling leads to development of numerous human diseases. In this study, we investigated the function of chromosome 7 open reading frame 41 (C7ORF41) in NF-*κ*B signaling during inflammation. C7ORF41 was upregulated in cells stimulated with tumor necrosis factor-alpha or lipopolysaccharide. Moreover, overexpression of C7ORF41 inhibited the activation of NF-*κ*B and decreased the expression of its downstream target genes. Notably, small hairpin RNA-mediated depletion of C7ORF41 increased the levels of downstream genes and enabled the activation of NF-*κ*B. In conclusion, C7ORF41 negatively regulated inflammation via NF-*κ*B signaling and p65 phosphorylation *in vitro*. These findings may help to diagnose and prognosticate inflammatory conditions and may help develop new strategies for the management of inflammation-related diseases.

## 1. Introduction

Inflammation is the first-line protective barrier in the host and is triggered by noxious stimuli [1]. However, aberrant activation of inflammation leads to the development of various diseases in patients if no proper therapeutic intervention was undertaken [2]. The three main signaling pathways involved in inflammation include nuclear factor- (NF-) *κ*B, Janus kinase/signal transducer and activator of transcription, and mitogen-activated protein kinase. NF-*κ*B has been well-characterized as a signaling pathway involved in eliciting an inflammatory response. NF-*κ*B is a large family of transcription factors containing five monomers in the NF-*κ*B network of mammals: RelA (p65), c-Rel, RelB, NF-*κ*B1 (p50/p105), and NF-*κ*B2 (p52/p100). The Rel homology domain is involved in binding DNA and dimerization. Among the related monomers, RelA (p65) has been studied in the greatest detail. Under normal conditions, NF-*κ*B dimers bind to the inhibitor of NF-*κ*B proteins (I*κ*Bs) and localize to the cytoplasm without exerting transcriptional activities. However, upon stimulation by intra- and extracellular factors, I*κ*B kinase (IKK) undergoes phosphorylation and ubiquitination, which triggers the degradation of I*κ*Bs and enables the nuclear shuttling of free NF-*κ*B [3, 4].

NF-*κ*B is activated at inflammation sites in various diseases and can regulate the expression of proinflammatory cytokines, synthesis of adhesion molecules, and transcription of more than 500 target genes associated with inflammation, proliferation, invasion, and cell survival [5, 6]. NF-*κ*B is present in endothelial cells and vascular smooth muscle cells in the atherosclerotic vascular wall. Activation of NF-*κ*B leads to the recruitment of a multitude of inflammatory cells and factors, such as tumor necrosis factor- (TNF-) *α*, interleukin-1*β* and interleukin-6, and interferon-*γ* [7]. Activated NF-*κ*B transcription factors can be found in synovial fibroblasts and *in vitro* models of the joints of animals and humans with rheumatoid arthritis [8]. Constitutively active NF-*κ*B signaling regulates the transcription of multiple genes involved in immune regulation, resulting in tumor cell proliferation, invasion, and evasion of apoptosis. An increasing number of NF-*κ*B signaling inhibitors have been shown to regulate these cellular processes. Because of serious adverse reactions, only a few have been approved for clinical use [9]. Recent studies have focused on the involvement of NF-*κ*B and other genes in the resolution of inflammation; in this process, one study identified the role of chromosome 7 open reading frame 41 (C7ORF41), which was discovered in 2013 [10] and has not been extensively studied.

C7ORF41, located on the short arm of human chromosome 7 (7p14.3), contains four exons and encodes a 131-amino acid-long protein. C7ORF41 has homologous genes in 246 species and encodes an evolutionarily conserved acidic protein whose function and domain organization remain unclear; it is heavily conserved in vertebrates and highly expressed in the brain, adipose tissues, kidneys, and ovaries [11, 12]. The ortholog of human C7ORF41 in Xenopus laevis, MTURN, plays an essential role in primary neurogenesis and regulates signaling pathways involved in neural differentiation [10]. The compound 12-O-tetradecanoylphorbol-13-acetate upregulates C7ORF41, induces megakaryocyte differentiation, and simultaneously activates NF-*κ*B signaling, thereby causing dynamic physiological changes [13]. Viral infection induces the upregulation of INKIT, which is an inhibitor of NF-*κ*B and IRF3 that restricts innate antiviral responses by depressing phosphorylation of p65 and IRF3 [14]. Therefore, we investigated the function of C7ORF41 in precursors of lineage-committed cells and hypothesized that C7ORF41 regulates a variety of signaling pathways. We focused on canonical NF-*κ*B signaling during inflammation and the mechanism(s) employed by C7ORF41 in inducing the inflammatory cascade. We identified C7ORF41 as a novel inhibitor of TNF-*α* or lipopolysaccharide- (LPS-) stimulated NF-*κ*B activity [15, 16].

## 2. Materials and Methods

### 2.1. Cell Culture

Mouse embryonic fibroblasts (MEF cells), p65-/-MEF cells, and human embryonic kidney HEK293T cells (kind gift from Prof. Zan Huang, School of Life Sciences of Wuhan University) were cultured in high-glucose Dulbecco's modified Eagle's medium (Gibco BRL, Grand Island, NY, USA), and human monocytic cells THP-1 were incubated in complete 1640 medium (Gibco BRL); both were supplemented with 10% heat-inactivated fetal bovine serum (Bio-one Biotechnology) and 1% streptomycin and penicillin (Life Technologies, Carlsbad, CA, USA). All cultures were passaged every 3 days.

### 2.2. Lentivirus Infection

The virus packaging cells HEK293T (5 × 10^6^) were inoculated into a 10 cm^2^ culture dish 1 day before transfection. Cells were transfected with the puromycin-resistant lentiviral vector and the packed plasmids (pSPAX2 and pMD2G). The medium was replaced with a fresh one 8 h later. After 40 h, the viral supernatant was collected from the transfected cells. THP-1 infection was performed by incubating cells with lentiviral supernatant in the presence of polybrene (5 *μ*g/ml) followed by centrifuging (1000 rpm) for 1 h. The infected cells were treated with puromycin for 7 days for subsequent experiments. To knock down C7ORF41, the sequences of short hairpin RNA (shRNA) oligonucleotides for human C7ORF41 were designed as follows: shC7-1: 5′-GCACTTCGAAATAGGACATCT-3′; shC7-3: 5′-GGCTCCCAGCATCCAAATTAA-3′. The shRNA oligonucleotide sequence for mouse C7ORF41 was shC7: 5′-GACGAGACGGCAGCTTACTTA-3′. To overexpress C7ORF41, phage-6tag-C7ORF41 was established.

### 2.3. RNA Extraction and Real-Time PCR

TNF-*α* (10 ng/ml) or LPS (100 ng/ml) was used to stimulate MEF cells, p65-/-MEF cells, and HEK293T for different times as indicated (MEF cells and p65-/-MEF cells: 0 h, 0.25 h, 0.5 h, 1 h, 4 h, and 8 h; HEK293T: 0 h, 1 h, 2 h, and 4 h). Total RNA was extracted using TRIzol (Invitrogen, Carlsbad, USA). A high-capacity reverse transcription kit (Promega, Madison, WI, USA) was used to synthesize cDNA. PCR was performed using a 7900HT Fast Real-Time PCR System (Applied Biosystems, Foster City, CA, USA) with an SYBR green one-step real-time PCR kit (Biotool, Houston, TX, USA), according to the manufacturer's instructions. The PCR conditions were as follows: one cycle of 15 min at 95°C, 40 cycles of 30 s at 95°C, 30 s at 60°C, and 1 min at 72°C. Primers were used for real-time PCR.

### 2.4. Dual-Luciferase Reporter Gene Assay

HEK293T cells were seeded into 24-well plates. After 24 h, cells were transfected with overexpressed (pHAGE-6tag-C7ORF41) or knockdown (pLenti-shRNA#1 or pLenti-shRNA#3) vectors with 100 ng of NF-*κ*B promoter fluorescent reporter plasmid and 2.5 ng of internal reference plasmid, phRL-TK. An empty lentiviral vector was used as a control. After 48 h of transfection, cells were stimulated with TNF-*α* for 6 h. Cells were then lysed, dissolved in luciferase assay buffer II, and centrifuged at 9729 ×g. The supernatant was collected, and the absorbance was measured with the Clarity Luminescence Microplate Reader (BioTek Instruments). The reaction was conducted with a dual-luciferase reporter gene detection kit (Promega, Madison, WI, USA) using the dual-luciferase reporter assay system (Promega).

### 2.5. Western Blot Hybridization Detection

MEF cells, p65-/-MEF cells, HEK293T, and THP-1 cells were harvested after stimulation with TNF-*α*/LPS for different times (MEF cells and p65-/-MEF cells: 0 h, 0.5 h, 2 h, 4 h, 8 h, 12 h, and 24 h; HEK293T: 0 min, 5 min, 10 min, 15 min, 30 min, and 45 min; THP-1 cells: 0 h, 0.5 h, 2 h, 4 h, 8 h, and 12 h), and 1 ml cell lysis buffer supplemented with PMSF and protease inhibitors was used for 10 × 10^6^ cells. Cells were lysed on ice for 30 min to 1 h. After centrifugation (12,000 ×g for 10 min), cell lysates were used for western blot. Total protein was separated on SDS-PAGE and transferred to a membrane. After blocking, and antibody incubation, the membrane was incubated with chemical reagents purchased from Sinopharm Chemical Reagent Co., Ltd. (Shanghai, China) following the manufacturer's instructions. Autography was performed for analysis.

### 2.6. Statistical Analysis

All experiments in this study were performed in triplicates for 3 times. Data was processed and analyzed using the GraphPad Prism software (GraphPad, Inc., La Jolla, CA, USA), and values were expressed as mean ± standard deviation. Differences between the two groups were calculated using a Student *t*-test (two-tailed, unpaired). *p* < 0.05 was considered significant and was marked as ∗; when *p* < 0.01, the difference was considered as extremely significant and marked as ∗∗; when *p* > 0.05, the difference was not considered as significant.

## 3. Results

### 3.1. C7ORF41 Is Associated with Inflammation

**Figure 1:**
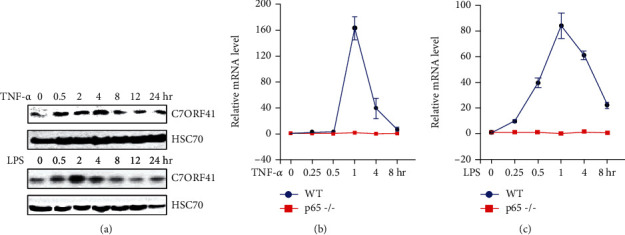
TNF-*α* or LPS induced C7ORF41 expression in MEF cells. (a) The protein level of C7ORF41 was measured using immunoblotting in mouse MEF cells treated with TNF-*α* or LPS for different times as indicated. (b, c) The mRNA level of C7ORF41 measured using quantitative RT-PCR in wild-type (WT) and p65 knockout (p65-/-) MEF cells treated with TNF-*α* (b) or LPS (c) for different times as indicated. Data are presented as the mean ± SD, *p* < 0.05; *n* = 3.

Important inflammatory stimulators include TNF-*α* and LPS. To test whether C7ORF41 may be involved in inflammation, we treated mouse embryonic fibroblasts with TNF-*α* or LPS for different times and measured C7ORF41 expression. We found that C7ORF41 was upregulated in response to TNF-*α* or LPS stimulation (Figure 1(a)). Particularly, protein levels of C7ORF41 peaked at 4 h after treatment with TNF-*α* and at 2 h after treatment with LPS; then, it gradually decreased during the next 4–8 hours. These results suggest that C7ORF41 is involved in the induction of inflammation (Figure 1(a)). Furthermore, we treated p65-/- MEF cells with TNF-*α*/LPS to determine whether NF-*κ*B was required for C7ORF41 upregulation during inflammation. Quantitative reverse transcription-polymerase chain reaction (RT-qPCR) showed that neither TNF-*α* nor LPS induced the expression of C7ORF41 in the absence of p65 compared to C7ORF41 expression in the wild-type (WT) cells. The mRNA levels followed a similar increasing pattern, peaking at 1 h after treatment with TNF-*α* or LPS and decreasing in WT cells with the protein levels of C7ORF41 (Figures 1(b) and 1(c)). Collectively, these findings demonstrate that C7ORF41 upregulation is associated with inflammation and mediated by p65. Our findings suggest a potential role of C7ORF41 in inflammation.

### 3.2. C7ORF41 Inhibits TNF-*α*-Induced NF-*κ*B Activation

**Figure 2:**
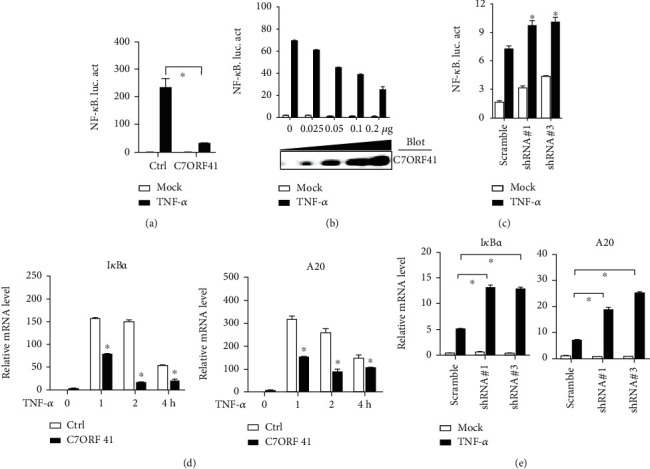
C7ORF41 inhibits TNF-*α*-induced activation of NF-*κ*B in HEK293T cells. (a) C7ORF41 overexpression decreased luciferase activity of the NF-*κ*B-responsive element. Data are presented as the mean ± SD; ^∗^*p* < 0.05, ^∗∗^*p* < 0.01, and ^∗∗∗^*p* < 0.001; *n* = 3; NS: nonsignificance compared to control cells. (b) Luciferase reporter assays analyzing NF-*κ*B promoter activity in HEK293T cells transfected with empty vector (Mock) or C7ORF41-encoding plasmid (0–0.2 *μ*g) for 20 h followed by treatment with TNF-*α* for 8 h. (c) C7ORF41 knockdown (shC7#1, shC7#3) increased luciferase activity of NF-*κ*B-responsive element. (d) mRNA levels of I*κ*B*α* (A) and A20 (B) were measured in C7ORF41 overexpressed and control (Ctrl) HEK293T cells treated with TNF-*α* for different times as indicated. (e) mRNA levels of I*κ*B*α* (A) and A20 (B) were measured in C7ORF41 knockdown (shC7#1, shC7#3) and control (Scramble) HEK293T cells treated with TNF-*α*.

To understand the role of C7ORF41 in inflammation, we first tested the effect of C7ORF41 function in the NF-*κ*B pathway that is a critical pathway involved in mediating inflammation. By using a dual-luciferase reporter system, we measured NF-*κ*B activity in the presence of C7ORF41 overexpression or downregulation. Notably, C7ORF41 upregulation drastically decreased the activity of NF-*κ*B in a concentration-dependent manner compared to control cells (Ctrl) (Figures 2(a) and 2(b)). In contrast, C7ORF41-depleted cells showed significantly lower activity of NF-*κ*B compared to control cells (Scramble) (Figure 2(c)). These findings suggest that C7ORF41 inhibits TNF-*α*-induced activation of NF-*κ*B.

Furthermore, we examined whether C7ORF41 is involved in suppressing NF-*κ*B target genes. We performed RT-qPCR to measure the levels of downstream target genes, I*κ*B*α* and A20. Upon treatment with TNF-*α*, the mRNA levels of I*κ*B*α* and A20 rapidly increased, peaking at 1 h in the control group. Similarly, C7ORF41 overexpression increased the mRNA levels of I*κ*B*α* and A20, peaking at 1 h; however, this increase was lower than that detected in control cells (Figure 2(d)). In contrast, C7ORF41 depletion increased the expression of I*κ*B*α* and A20 compared to control cells (Figure 2(e)). These results indicate that C7ORF41 inhibits TNF-*α*-induced activation of NF-*κ*B.

### 3.3. C7ORF41 Suppresses the IKK*α*/*β*-I*κ*B*α*-p65 Axis in TNF-*α*-Induced Activation of NF-*κ*B Signaling

**Figure 3:**
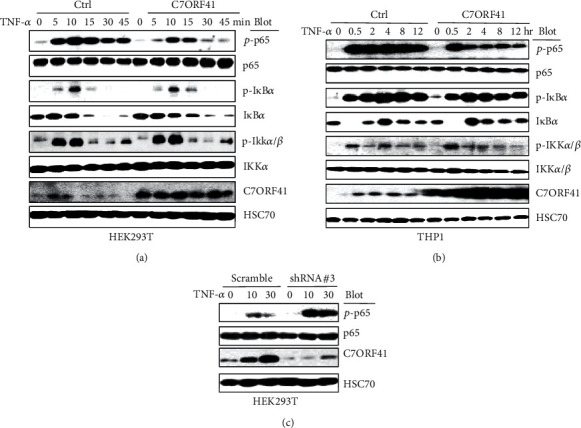
C7ORF41 inhibits TNF-*α*-induced activation of the NF-*κ*B signaling pathway in HEK293T and THP-1 cells. (a, b) Control (Ctrl) or C7ORF41-overexpressing HEK293T (a) or THP-1 (b) cells were stimulated with TNF-*α* for the indicated times. The phosphorylation of p65 (p-p65), I*κ*B*α* (p-I*κ*B*α*), and IKK*α*/*β* (p-IKK*α*/*β*) and the expression of p65, I*κ*B*α*, IKK*α*/*β*, and C7ORF41 were detected using western blotting. (c) Scramble or C7ORF41 knockdown (shRNA#3) HEK293T cells were stimulated with TNF-*α* for the indicated times. Phosphorylation of p65 (p-p65) or expression of p65 and C7ORF41 was detected using western blotting. HSC70 was used as a control.

To confirm the effect of C7ORF41 on NF-*κ*B signaling, we overexpressed or downregulated C7ORF41 in HEK293T cells and treated cells with TNF-*α* for different times. We performed western blotting using total proteins isolated from the transfected cells to further analyze C7ORF41-mediated inhibition of TNF-*α*-induced NF-*κ*B activation in cells stimulated with TNF-*α* for different times. The protein levels of p-IKK*α*/*β*, p-I*κ*B*α*, I*κ*B*α*, and p-p65 were significantly increased (at 5–10 min) and subsequently decreased in control HEK293T cells. However, the increased levels of p-I*κ*B*α* and p-p65 were repressed by C7ORF41 overexpression (Figure 3(a)). In fact, C7ORF41 retained I*κ*B*α* expression as a repressor of p65. The total protein levels of IKK*α*/*β* and p65 were not significantly changed in both groups. Similar results were observed in THP1 cells that protein levels of p-IKK*α*/*β*, p-I*κ*B*α*, and p-p65 were significantly increased in THP-1 control cells stimulated with TNF-*α* (at 30 min); then, the overexpression of C7ORF41 suppressed the levels of p-I*κ*B*α* and p-p65 (Figure 3(b)). In contrast, the depletion of C7ORF41 enhanced the p-p65 levels and activated NF-*κ*B signaling in HEK293T cells (Figure 3(c)). Collectively, our results strongly suggest that C7ORF41 inhibits TNF-*α*-induced NF-*κ*B signaling by regulating p65 phosphorylation in HEK293T and THP-1 cells.

## 4. Discussion

In this study, we explored the function of C7ORF41 in mouse and human cell lines and found that C7ORF41 regulated inflammation. C7ORF41 was upregulated during inflammation and inhibited the activation of NF-*κ*B signaling by reducing the phosphorylation of p65 in HEK293T and THP-1 cells. Numerous transcriptional regulators that prevent NF-*κ*B signaling are involved in cytokine-induced NF-*κ*B activation and inflammation *in vitro* and *in vivo*. These regulators function in the various phases of NF-*κ*B signaling, such as the interactions between the subunits of I*κ*B kinase [17], phosphorylation of p65/RelA [18], and nuclear shuttling of NF-*κ*B. We focused on C7ORF41 because of its unknown characteristics and the need to understand the molecules involved in inflammation. Therefore, we investigated the mechanism(s) employed by C7ORF41 in regulating the inflammatory response. First, we successfully established an in vitro cell model for classical inflammation stimulated by TNF-*α* and LPS [15, 16]. We observed a p65-dependent increase in the expression of C7ORF41 during inflammation. A previous study showed that p65 plays a central role in NF-*κ*B signaling [19]. This indicates the importance of C7ORF41 in NF-*κ*B signaling.

Second, using C7ORF41-overexpressing or C7ORF41-depleted cells, we observed that increasing concentrations of C7ORF41 inhibited the activation of NF-*κ*B, thereby negatively affecting inflammation. This inhibition was mediated by reduced phosphorylation of p65. Our preliminary experiments suggest that C7ORF41 interacts with p65 (data not shown). However, the detailed mechanism by which C7ORF41 regulates NF-*κ*B signaling remains to be dissected.

NF-*κ*B is a complex transcription factor that regulates gene expression, inflammation, and signal transduction [20]. NF-*κ*B has also been reported to be involved in tumorigenesis, although the underlying mechanisms remain unclear [21, 22]. Recent studies have focused on NF-*κ*B signaling inhibitors because of the importance of inflammation in the development of multiple diseases [17]. However, these studies have not addressed the applications of these inhibitors in real-life scenarios, such as clinical trials [23, 24]. Therefore, there is an urgent need for detailed studies on the molecular mechanisms involved in inflammation to increase the understanding and facilitate treatment of various diseases as well as provide a platform for characterizing other targets that can inhibit NF-*κ*B signaling; these targets can be used to develop clinical applications, such as targeted therapy [25, 26].

Whether C70RF41 may serve as a potential therapeutic target of inflammation remains unclear. Further studies are needed to determine the utility of this target for treating acute and chronic diseases, and tumors associated with inflammatory lesions. Studies have reported aberrant expression of C7ORF41 during the early stages of human embryonic development, particularly in nerve cell differentiation [27]. Samples from the brain and cerebrospinal fluid of patients with autism spectrum disorders show increased contents of inflammatory molecules, such as IL-1*β*, IL-6, TNF-*α*, MCP-1, and CXCL8/IL-8 [28–30]. Pathological specimens from these patients reveal active microglial proliferation and increased concentrations of activated inflammatory factors [31]. Therefore, C7ORF41 may also play a regulatory role in neuroimmunity. The potential of C7ORF41 for treating neuronal developmental disorders and regulating megakaryocyte differentiation and neural cell differentiation must be assayed in the future. Further studies are warranted to gain a greater understanding of the clinical potential of C7ORF41. As the findings presented in this study were based on *in vitro* cell models, future research will focus on evaluating whether C7ORF41 can effectively reduce the inflammatory response *in vivo*, and via signaling pathways other than NF-*κ*B.

## 5. Conclusions

C7ORF41 negatively regulated inflammation via suppressing p65 phosphorylation *in vitro*. These findings may be useful for developing new strategies for the management of inflammation-related diseases.

## Data Availability

The data used to support the findings of this study are available from the corresponding author upon request.
